# The Therapeutic Potential of Inflamed Gingiva-Derived Mesenchymal Stem Cells in Preclinical Studies: A Scoping Review of a Unique Biomedical Waste

**DOI:** 10.1155/2021/6619170

**Published:** 2021-02-10

**Authors:** Gamilah Al-Qadhi, Sarah Al-Rai, Layla Hafed

**Affiliations:** ^1^Department of Basic Dental Sciences, Faculty of Dentistry, University of Science and Technology, Yemen; ^2^Department of Conservative and Preventive Dentistry, Faculty of Dentistry, Saba University, Yemen; ^3^Department of Oral Pathology, Faculty of Oral and Dental Medicine, Ahram Canadian University, Giza, Egypt

## Abstract

Searching for considerable abundance, simple, and accessible sources in stem cell-based therapy opens the door for isolation of a new population of oral/dental stem cells known as inflamed gingiva-derived mesenchymal stem cells, which have recently come to light with promising therapeutic potential in tissue regenerative therapy. Following the Preferred Reporting Items for Systematic reviews and Meta-Analyses extension for Scoping Reviews guidelines, this scoping review is aimed at highlighting the possible therapeutic potential of inflamed gingiva-derived mesenchymal stem cells in preclinical studies carried out to date and presenting the current evidence depends upon their comparison to the healthy gingiva-derived mesenchymal stem cells or other mesenchymal stem cell sources. A comprehensive electronic search using (PubMed, Embase, Scopus, and Web of Science) databases and a manual search of relevant references were conducted until June 2020. Included studies were assessed using a combination tool, including the guidelines for reporting preclinical *in vitro* studies on dental materials, which were based on the modification of the Consolidated Standards of Reporting Trial checklist and the guidelines for animal research: reporting of *in vivo* experiments. The initial research provided 360 articles, with 13 articles that met the inclusion criteria. While most of the included studies lacked randomization, blinding, and sample size calculation, they were designed accurately in other aspects of the guidelines. The results of this scoping review indicated that inflamed gingiva-derived mesenchymal stem cells could be effective in terms of osteogenic differentiation, collagen fiber formation, immunoregulation, migration capacity, and testing of dental material and may present a reliable alternative source for healthy gingiva-derived mesenchymal stem cells.

## 1. Introduction

Stem cell-based therapy has attracted many researchers in the regenerative medicine field, primarily mesenchymal stem cells (MSCs), which play a fundamental role with the surrounding microenvironment during the regenerative process [1]. Bone marrow and adipose tissues are the most commonly used sources. However, the associated invasive surgical procedure for both patient and donor opens the door to look for easily accessible and less invasive alternative sources. The discovery of prevalent cells having the typical characteristics of MSCs in the oral cavity has made scientists become more interested in oral tissues. Oral-derived mesenchymal stem cells can be easily isolated from diverse sources, including dental pulp of permanent teeth or exfoliated deciduous teeth, periodontal ligaments, dental follicle, apical papilla, and alveolar bone. These cells displayed great plasticity towards trilineages, particularly towards the osteogenic lineage [2].

Moreover, gingival mesenchymal stem cells (GMSCs) are one of these doors denoting the recent newly identified population from dental/oral tissue. In several studies, mesenchymal stem cells isolated successfully from gingiva and met the minimal criteria proposed by the International Society for Cellular Therapy (ISCT) for MSCs' characterization. Human GMSCs showed self-renewal capabilities via the formation of colony-forming units (CFU), trilineage differentiation ability into osteoblast, adipocyte, and chondrocyte cell lineage under *in vitro* culture condition and expression of MSC markers and stem cell-specific genes [3–5].

Interestingly, the discarded diseased gingival tissue might represent a possible valid alternative source to healthy ones. Mesenchymal stem cell-like population obtained from inflamed gingival tissue had similar functional properties to the GMSCs isolated from healthy tissue. The trilineage differentiation capacity and surface marker expression were similar in both populations. Additionally, ectopic transplantation of stem cells from both sources resulted in the formation of a connective tissue-like structure similar to natural gingiva. However, the doubling time was declined in GMSCs derived from inflamed tissue [6, 7].

Likewise, other MSCs of inflamed dental origin were successfully isolated and characterized, and the MSCs isolated from inflamed gingiva and dental pulp tissues displayed a higher proliferation rate than the healthy control. Particularly, proinflammatory cytokines such as interleukin-1*β* (IL-1*β*) and tumor necrosis factor-*α* (TNF-*α*) promoted the expansion of both sources *in vitro* culture. The osteogenic markers were significantly higher in proinflammatory cytokine-treated dental pulp or gingival tissue in comparison to their untreated healthy ones. Clearly, the inflammatory environment did not change MSC markers or numbers neither osteogenic differentiation [8]. Further, the differentiation capacity of GMSCs and periodontal ligament mesenchymal stem cells (PDLSCs), whether derived from healthy or diseased periodontal ligament tissues, was similar [9].

On the contrary, another study stated that MSCs derived from inflamed periodontal ligament exhibited impaired immunomodulatory function and immune response via inhibition of T cell proliferation along with enhancement of osteoclastogenesis and alveolar bone loss in periodontitis, while cells obtained from healthy tissues showed the normal profile [10].

Furthermore, when GMSCs and PDLSCs were cultured in an osteogenic induction medium supplemented with TNF-*α* and IL-1*β* to create an inflammatory microenvironment, both cells displayed a decline in the formation of mineralized nodules, alkaline phosphatase (ALP) activity, and expression of the following osteogenic markers: osteocalcin (OCN), runt-related transcription factor 2 (RUNX2), and collagen type 1 (COL1) [11].

Recently, GMSCs from healthy and inflamed tissue were loaded on microperforated membranes to evaluate their proliferation and migration capacity. The finding revealed that both sources are functionally and phenotypically characterized as MSCs. Notably, the proliferation rate was significantly higher in inflamed GMSCs. Concerning the migration dynamics, GMSCs whether from healthy or inflamed tissues exhibited a similar migration pattern toward chemoattractant through smaller pore sizes. It is worth noting that a better migratory activity was reported in GMSCs derived from healthy tissues in the case of large pore-sized membrane [12].

Similarly, the type of scaffold could play a critical role in cell growth and lineage direction. For example, GMSCs obtained from diseased tissue and seeded on two types of the scaffold, nano hydroxyapatite (HA) and collagen type 1, revealed that collagen type 1 scaffold supported growth and osteodifferentiation of GMSCs more than a nano HA [13]. In addition to the self-renewal, multipotent differentiation and migration capacity, which collectively represent one of the GMSC mechanisms in the regenerative approach, GMSCs have significant immunomodulatory and anti-inflammatory functions. The latter mechanism is mediated through suppression of inflammatory infiltrates and proinflammatory cytokines, induction of immunosuppressive factors such as IL-10, and increased infiltration of regulatory T cells [3]. Along with that, GMSCs promoted polarization of macrophages toward the anti-inflammatory M2 phenotype via increased expression of IL-10 and decreased expressions of IL-6 and TNF-*α* [14].

Additionally, Toll-like receptors (TLRs) may be responsible for cross talk between GMSCs and their inflammatory environment. Fawazy El-Sayed et al. [15] investigated the TLR expression profile of GMSCs in healthy and diseased conditions. The results showed that GMSCs cultured in basic medium expressed the following TLRs: 1, 2, 3, 4, 5, 6, 7, and 10, while those cultured in medium supplemented with inflammatory cytokines like IL-1*β*, interferon-*γ* (IFN-*γ*), TNF-*α*, and IFN-*α* significantly expressed TLRs 1, 2, 4, 5, and 7 as well as 10 without TLR 6. This expression may determine the therapeutic potential of GMSCs in recognizing the pathway of pathogen-associated molecular patterns (PAMPs) in periodontal disease.

GMSCs regardless of their origin (healthy or inflamed gingival tissue) exhibited immunoregulatory functions, particularly immunosuppression, in a mouse skin allograft model through upregulation of putative systemic regulatory T cells (Tregs) [6]. On the contrary, MSCs derived from inflamed gingiva revealed impaired stemness and deficient immunomodulatory function. Fortunately, this deficiency could be rescued by pretreatment of inflamed GMSCs culture with acetylsalicylic acid (ASA), a type of nonsteroidal anti-inflammatory drug (NSAID), which was able to increase the expression of Fas Ligand (FasL) and, in turn, induce T cell apoptosis [16]. Overall, the finding of I-GMSCs is a significant step in tissue regeneration as the existence of gingival tissue within a microenvironment characterized by constant bacterial challenges and inflammatory changes. In addition to that, their ability to resist these changes with maintaining their MSC's features opens the door for the possibility of using this source in the *in vivo* regenerative applications, where similar inflammatory factors are involved in a regenerative process [17].

The initial inflammation in the periodontal tissues is a physiologic rather than a pathological mechanism, and it is mainly characterized by plaque formation [18]. Although the oral rinses have antiplaque and antimicrobial actions against plaque accumulation [19], their effects on osteoblast precursors should be taken into consideration. In *in vitro* study, mouth rinses inhibited cell viability and changed the morphology of osteoblastic precursor cells regardless of their type, duration, or alcohol content. However, this finding was limited and not sufficient to give evidence since it was confined to a laboratory environment, and further *in vivo* studies and clinical trials are required to assess the safety of oral rinse on various progenitor cells [20].

Inflammatory microenvironments that characterize different oral pathologies such as periodontitis and periapical cyst have shown that they are capable of modifying the characteristic of MSCs and even enhancing them in certain cases. For instance, MSCs obtained from human periapical cysts exhibited similar features to oral-/dental-derived MSCs such as high proliferative rate and extensive multipotency which makes them a potential source in regenerative medicine [21].

Stem cells isolated from the discarded gingival tissue have received much attention over recent years. The effectiveness of any intervention should be excessively examined in preclinical studies before translating into a clinical setting. To date, there was no available scoping review mapping the effectiveness of inflamed gingiva-derived mesenchymal stem cells in the tissue regenerative field. Therefore, the purpose of the current scoping review was to present what is known from the previous literature about the therapeutic role of inflamed GMSCs and certainly addressed the following question: To what extent would I-GMSCs be considered as an alternative approach for MSCs derived from healthy tissue in preclinical studies? The PICO relevant key elements were set as follows: population, in vitro cultures and animal studies; intervention, inflamed GMSCs; control or comparator, control: healthy GMSCs or other healthy MSC sources and/or comparator: other inflamed MSC sources; outcome, therapeutic potential.

## 2. Materials and Methods

The current scoping review was conducted following the Preferred Reporting Items for Systematic reviews and Meta-Analyses extension for Scoping Reviews (PRISMA-ScR) guidelines [22].

### 2.1. Protocol Registration

he protocol was registered with the Open Science Framework for scoping review protocol registration on 15 September 2020 at the following link: https://osf.io/jt62m and registration DOI: 10.17605/OSF.IO/JT62M.152.

### 2.2. Eligibility Criteria

The following articles, regardless of their outcome measures, were selected to be included in the current scoping review: (a) original experimental studies comparing GMSCs from the diseased tissue versus those isolated from healthy ones or those obtained from other MSC sources, (b) studies that treated GMSCs with proinflammatory cytokines in culture versus untreated cells, and (c) preclinical studies (*in vitro* or *in vivo*) and there was no language or date restrictions. On the other hand, nonexperimental articles such as reviews, case reports, and expert opinions were excluded during the first phase of screening. Further, studies using derivatives of inflamed GMSCs, such as exosomes or microvesicles, and studies that applied genetic modification were excluded during the second phase of screening. The materials and methods section should contain sufficient detail so that all procedures can be repeated. It may be divided into headed subsections if several methods are described.

### 2.3. Information Sources and Search Strategy

In June 2020, four electronic databases (PubMed, Embase, Scopus, and Web of Science) were searched without any date or language restrictions. Key terms such as inflamed gingiva OR diseased gingiva OR discarded gingiva OR hyperplastic gingiva derived mesenchymal stem cells OR inflammation-derived gingival stem cells were initially used and resulted in a few retrieved papers. Therefore, a combination of keywords and index term keywords was expanded to include the following items: inflammation OR inflamed OR diseased OR discarded OR hyperplastic AND gingival mesenchymal stem cell OR gingiva derived mesenchymal stem cell OR gingival tissue-derived mesenchymal stem cells OR gingiva derived stromal cell OR multipotent gingival stromal cell OR multipotent gingiva progenitor cell OR gingiva stem cells AND animal model OR experimental animal OR laboratory animal OR *in vivo* study OR *in vitro* study OR *in vitro* OR *in vitro* technique OR cell culture technique OR cell culture method OR culture technique OR preclinical study. The search strategy for each database was documented, and the retrieved references were combined and saved in one Mendeley folder. Then, duplicate records were removed.

### 2.4. Selection of Sources of Evidence

The selection of studies was carried out independently through two phases by two authors (Al-Qadhi and Hafed). First, the title and abstract of retrieved papers were screened to identify potentially eligible papers. References that did not fulfill the inclusion criteria were excluded. Second, full-text articles were screened in detail to assess and decide which study was appropriate for inclusion. Any disagreement in study selection was resolved by discussion, and the third author (Al-Rai) asked for her opinion. Reasons for exclusion were identified and documented.

### 2.5. Data Charting Process and Data Items

All authors participated in the process of designing the initial table and determining the data items. Data from the relevant studies were charted into a predesigned table by one author (Al-Qadhi) and then checked by two authors (Hafed and Al-Rai). Similar to the previous step, any disagreement in data entry was resolved by discussion with team members. The following items were extracted: study ID (author and year of publication), characteristics of methodology (study design; *in vitro* or *in vivo*, source of stem cells, sample collection, experimental sets or groups, scaffold, or carrier if present), and results of the included studies (clonogenic ability, population doubling capacity, phenotypic profile, differentiation profile, outcome measures, method of investigation, and endpoint).

### 2.6. Critical Appraisal of Individual Sources of Evidence

The quality assessment of included studies was carried out independently by (Al-Qadhi and Al-Rai). It should be noted that there is not a defined grading quality scale for preclinical studies, particularly *in vitro* studies. The experimental design of all included studies was *in vitro* or *in vitro* followed by *in vivo* (ectopic confirmation). Therefore, guidelines for reporting preclinical *in vitro* studies on dental materials, which based on the modification of the Consolidated Standards of Reporting Trial (CONSORT) checklist [23], and the guidelines for Animal Research: Reporting of In Vivo Experiments (ARRIVE) were applied with some modification for quality assessment of included studies [24].

The grading system had two forms of responses. The first form was as follows: clearly inadequate, possibly accurate, and clearly accurate responses, which were scored with (1), (2), and (3), respectively; the second form was yes or no, which were scored with (0) and (1). The rating of overall quality was as follows: 1-9: low, 10-19: moderate, and 20-29: high for *in vitro* studies and 1-12: low, 13-24: moderate, and 25-38: high for *in vitro* followed by *in vivo* studies.

### 2.7. Synthesis of Results

The findings of the current research were summarized and presented in a tabular and textual qualitative manner. Meta-analysis was not applicable in the scoping review.

## 3. Results

### 3.1. Selection of Sources of Evidence

A total of 359 citations was identified from the defined electronic databases (PubMed = 181, Embase = 25, Scopus = 143, and Web of Science = 10), and one additional relevant article was detected through the manual search. After removing duplicate records, 260 papers were identified and subjected to the first level of screening (titles and abstracts) in which 240 irrelevant papers were excluded.

**Figure 1:**
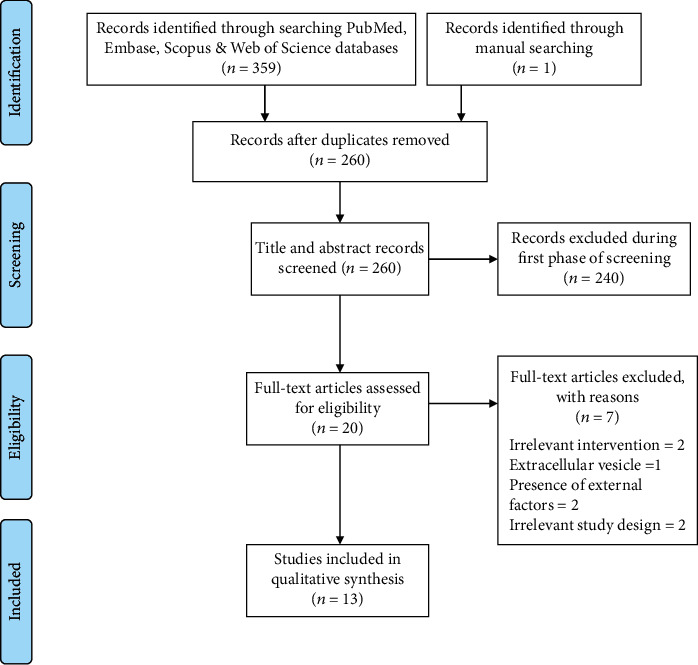
Flow diagram showing the different phases of literature screening for the scoping review process. (Editable file: PRISMA Flow Diagram, Liberati et al., 2009).

At the second level of screening (full texts), the remaining 20 articles were screened further and assessed for eligibility. Of these, seven articles were excluded as they did not meet the inclusion criteria. In detail, one study used stem cells from inflamed periodontal ligaments, not from the gingiva [9], one study did not use the inflamed gingival source (irrelevant intervention) [25], and one study used inflammatory cytokine-treated cells without untreated or healthy control groups [26]. Correspondingly, three studies used extracellular vesicles or external factors including microvesicles [27], epigenetic modification [28], and Retinol [29], as well as one study used stem cells obtained from healthy gingiva in an inflammatory animal model [30]. Thus, 13 studies were included in the current scoping review (Figure 1).

### 3.2. Characteristics of Sources of Evidence

The characteristics and the results of the included studies are reported in Tables 1 and 2, respectively. The included studies were published between 2011 and 2020. The experimental design of all studies was *in vitro* [6–8, 11–13, 15, 16, 31–35], and five of these were used *in vivo* models for further assessment [6, 7, 11, 16, 32]. All studies used the human as MSC source except one study used mice [16].

The majority of studies provided information about the diseased tissue sampling, while one article did not report the details [12]. MSCs from diseased gingiva were collected, whether directly during the gingivectomy procedure, extraction of periodontally affected teeth, and flap debridement [6–8, 13, 31, 32, 35] or indirectly via inflammatory preconditioned culture. In particular, GMSCs were treated with a culture containing inflammatory cytokines such as TNF-*α*, IL-1*β*, IFN-*γ*, IFN-*α*, and IL-3 [11, 12, 15, 33], and an inflammatory animal model was created in one study, ligature-induced periodontitis in mice [16]. Regarding the healthy source, MSCs from healthy gingiva were obtained during the normal dental processes such as crown lengthening procedure and extraction of third molars due to impaction or orthodontic treatment.

In detail, five studies used H-GMSCs versus I-GMSCs [12, 13, 16, 31, 35], and two studies compared H-GMSCs with I-GMSCs as well as bone marrow mesenchymal stem cells (BMSCs) [6] or PDLSCs [7]. Further, two articles compared inflammatory cytokine-treated H-GMSCs with untreated ones [15, 33], and two studies compared inflammatory cytokine-treated H-GMSCs to untreated ones, as well as inflammatory cytokine-treated BMSCs and adipose-derived stem cells (ADSCs) [31], PDLSCs [11], and dental pulp stem cells (DPSCs) [8, 32].

The majority of studies performed several assessments regarding the characteristic of interventions, including clonogenic potential, proliferation rate, phenotypic profile, and functional differentiation (osteogenic, adipogenic, and chondrogenic [6, 7, 12, 16, 35], osteogenic and adipogenic [31, 32], and osteogenic [13]) for MSCs derived from both healthy and diseased gingival tissue. Three studies performed the characterization for MSCs derived from healthy tissue only [11, 15, 33], and one article did not conduct any MSC characterization assays [31] (Table 2).

A variety of outcomes in terms of the therapeutic potential of inflamed gingiva-derived MSCs was reported using appropriate methods of investigation. It should be noted that some studies have more than one outcome. These outcomes included osteogenic potential [8, 11, 13, 31–33], migration capacity [12, 34], formation of collagenous tissue [6, 7, 32], testing biocompatibility of resin composite [32], immune-regulation including TRL expressions [15], immunomodulation [6], and inflammatory receptor expression [31].

### 3.3. Critical Appraisal within Sources of Evidence

Overall, most of the included studies provided adequate information about abstract, background, objectives, the ethical approval, study design, experimental groups, result and its interpretation, outcome evaluation, statistical method, funding source, conflict of interest, and peer-review publication, following the items of the appropriate guideline. However, the majority of them lacked sample size calculation and provided incomplete details about replication, randomization, and blinding. All studies, with the exception of study conducted by Tomasello et al. [8], did not explain any excluded data, such as how many samples processed successfully or unsuccessfully due to bacterial contamination or other factors?. For *in vivo* part, none of the included studies stated the required information about 289 housing, husbandry conditions, the baseline characteristics, and the health status of animals. The quality of the *in vitro* and *in vivo* studies selected in this scoping review is shown in supplementary material I.

### 3.4. Results of Individual Sources of Evidence

#### 3.4.1. Clonogenic, Proliferation, and Population Doubling Capacities

The clonogenic ability of I-GMSCs was higher than H-GMSCs in four studies [6, 8, 13, 31], lower in two studies [16, 33], and no significant difference between the two sources in three studies [7, 12, 32]. Likewise, the proliferation capacity of I-GMSCs was faster than H-GMSCs in four studies [7, 8, 13, 32] and lower in two studies [16, 33], and four articles reported that both I-GMSCs and H-GMSCs exhibited an increase in proliferation rate, somewhat similar to each other [6, 12, 31, 35]. It should be mentioned that two studies carried out the previous analysis for healthy tissue only [11, 15], and another two studies did not do this investigation at all [34, 35]. In the light of doubling population capacity, I-GMSCs were higher than H-GMSCs in four studies [6, 8, 12, 13] and shorter in two studies [7, 16]. The remaining studies did not conduct this assay (Table 2).

#### 3.4.2. MSC Characterization

Concerning the specific features of MSCs, both I-GMSCs and H-GMSCs had typical MSC-associated surface markers, a similar positive expression for MSC markers STRO-1, CD29, CD44, CD90, CD105, and CD146 and negative for CD34 and CD45 in six studies [6, 7, 12, 32–34]. However, two studies reported higher expression of positive markers in diseased groups than healthy controls [8, 13], while one study stated the opposite [16]. Similarly, in five articles, both I-GMSCs and H-GMSCs had a similar trilineage differentiation capacity: osteogenic, chondrogenic, and adipogenic [6, 7, 12, 35] and osteogenic differentiation only [13]. Different capacity was reported in four articles, in which I-GMSCs showed lower osteogenic and adipogenic capacities [16, 32] and decline osteogenic potential [11], and H-GMSCs had higher osteogenicity, while I-GMSCs had a higher adipogenesis [33] (Table 2).

#### 3.4.3. Main Findings of the Outcome

The following outcomes reported in the selected studies are as follows:

*(1) Osteogenic Differentiation*. Six of the included studies showed that I-GMSCs had potential osteogenic differentiation [8, 11, 13, 31–33]. More specifically, inflammatory cytokine-treated H-DPSCs and H-GMSCs as well as I-GMSCs were deeply stained and highly expressed osteogenic markers than untreated H-MSCs [8]. Although both cell sources had a tendency toward osteogenic differentiation, H-GMSCs had higher osteogenicity, while I-GMSCs showed increased adipogenesis [33]. Likewise, both sources showed a moderate expression of osteogenic markers (RUNX2, OCN, and osteopontin (OPN)) [13]. It is worth noting that the selective anti-inflammatory intervention could enhance osteogenic differentiation of GMSCs [35].

Despite the fact that I-GMSCs showed a decline in the osteogenic differentiation [11, 32], particularly after treatment with inflammatory cytokines [34], GMSCs had more resistance to inflammation-related changes in terms of osteogenic potential *in vitro* and bone formation *in vivo* compared to PDLSCs [11].

*(2) Formation of Collagenous Tissue*. Three of the included articles confirmed that the I-GMSCs formed a dense connective tissue similar to normal gingiva. Both inflamed and noninflamed sources generated dense collagenous connective tissue [6, 7, 32]. In particular, type 1 collagen [6, 32] and expression of intrinsic cytokines as matrix metallopeptidase-1 and -2 (MMP-1 and -2), IL-1, IL-6, and TNF-*α* were more evident in I-GMSCs [34].

*(3) Immunoregulation*. Concerning the immunoregulation, two studies demonstrated this function. Both GMSC sources (inflamed and healthy) showed distinct immunoregulatory functions in the allograft mouse model through elevation of Tregs with comparison to BMSCs [6]. H-GMSCs expressed TLRs 1, 2, 3, 4, 5, 6, 7, and 10, while GMSCs treated with inflammatory cytokines significantly expressed TLRs 1, 2, 4, 5, 7, and 10 without TLR 6 [15].

*(4) Migration Capacity*. Two of the selected studies reported that I-GMSCs migrated efficiently. No significant difference was observed between I-GMSCs and H-GMSCs, considering that IL-3 enhanced migration, motility, and wound closure; yet, BMSCs and ADSCs showed a higher expression of IL-3R*α* than GMSCs [31]. Notably, H-GMSCs migrated better than I-GMSCs through large-pore membrane [12].

**Figure 2:**
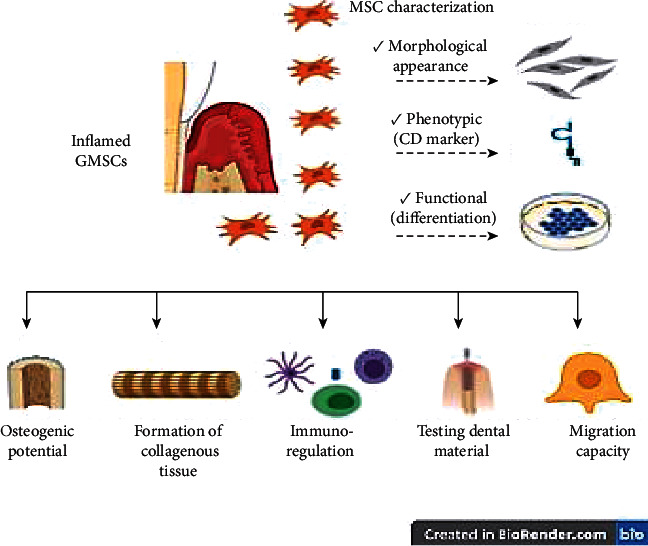
Possible applications and therapeutic roles of inflamed gingiva-derived mesenchymal stem cells.

*(5) Testing Biocompatibility of Resin Composite*. The study by Soancă et al. [32] is the only study that assessed the behaviour of I-GMSCs in relation to some commercial resin dental composite. This study suggested that I-GMSCs can be used as a useful and valued cell line for testing dental materials (Table 2 and Figure 2).

### 3.5. Synthesis of Results

Since the current scoping review included outcome measures that vary considerably, we focused on describing the included studies qualitatively in terms of their results rather than meta-analysis.

## 4. Discussion

The purpose of the current study was to review the potential therapeutic role of inflamed gingiva-derived mesenchymal stem cells in preclinical studies and highlight their role in comparison to healthy gingiva or other MSC sources. The preclinical experiment is the basic building block of any novel therapeutic approach. The majority of *in vitro* included studies fulfilled items related to the presenting and interpreting the data and used appropriate statistical analysis. However, the methodological quality assessment revealed a lack of important items like sample size calculation, randomization, blinding, and explanation of any excluded data.

It is thought these items can be applied for clinical and *in vivo* studies, and they are not practical for *in vitro* studies. Since many simple procedures should be added to design *in vitro* studies in order to reduce the bias, a well-designed *in vitro* experiment will positively affect their outcome and, in turn, their translation to *in vivo* and clinical applications. Randomization is essential for *in vitro* experiments because it deals with various issues that do not always have the same environment. For this reason, a culture flask or dish can be assigned at random to intervention and a column of wells in a multiwell plate may all be allocated to the same group. Thus, the column is considered as a single experimental unit, and the statistical analysis should be performed on the whole data from the whole column rather than on data from individual wells [23, 36]. Additionally, selecting fields for capturing histological or radiographical pictures should be done at random as well.

Before data processing, it might be useful to use a sample coding to blind the treatment category. Moreover, sample size can be estimated using either power analysis or the resource equation method, and the latter one is suitable in the case of *in vitro* studies where the absence of ethical issue and somewhat inexpensive experimental units so the upper limit can be increased [36]. The analysis of *in vivo* studies revealed several issues similar to the *in vitro* studies, namely, sample size, randomization, and blinding. In addition to that, all studies did not have enough and sufficient information about housing and husbandry conditions of animals, baseline data (characteristic and health status of animals), and an explanation of any excluded data. Determination of sample size is one of the important steps in designing animal studies; a fewer number of animals may lead to missing of any significant difference, and more number of animals selected may lead to misuse of resources and ethical problems [37].

Also, the standardization process is required to accurately evaluate the behaviour of cells. Recently, an innovative method using computer-aided design (CAD) technology was introduced to design a standardized sample culture plate model for *in vitro* studies to provide similar size and shape of scaffolds, to ensure optimum interaction between the seeded cells and these biomaterials and, in turn, to reduce bias in the measurements and results [38].

In respect of clonogenic, proliferation, and doubling population capacities, most of the selected studies indicate that there was no significant difference between I-GMSCs and H-GMSCs; even the inflamed group was higher than the healthy one indicating that the inflamed condition could positively affect the regeneration potential of GMSCs. It has to be mentioned that not all included studies performed the previous assay so we present the available finding. Equally important, the majority of articles show that both I-GMSCs and H-GMSCs display a positive expression of the principle MSC markers: STRO-1, CD29, CD44, CD90, CD105, and CD146 and negative expression of hematopoietic markers: CD34 and CD45. Both sources have the ability to differentiate into osteogenic, chondrogenic, and adipogenic lineage, while a minority of studies indicate that I-GMSCs have lower osteogenic and adipogenic differentiation capacity than the healthy groups. This finding is consistent with minimal criteria for MSCs proposed by the International Society for Cellular Therapy (ISCT) [39], so the resistance of GMSCs to inflammatory stimuli and maintaining their MSC features under inflammatory condition make them an attractive MSC source in tissue regenerative researches, where they could be subjected to similar inflammatory microenvironment [17].

Overall, the osteogenic potential and formation of collagenous tissues are confirmed by histological, immunohistochemical, and gene expressions. The most common methods used to evaluate the osteogenic potential of included studies are Alizarin Red staining, Haematoxylin and Eosin (H&E) staining, and the expression of osteogenic markers (RUNX2, ALP, OCN, and OPN). The immunohistochemical analysis used to assess the collagenous tissue formation has been reported by the studies conducted by Tang et al. and Ge et al. [6, 7]. In one study, H-GMSCs had higher osteogenicity, while I-GMSCs showed increased adipogenesis and this is maybe accounted for the difference in epigenetic factors that are associated with chronic periodontitis and affect the differential directions of these cell lines [33]. Several included studies confirmed that osteoblastic differentiation capacity under proinflammatory treated culture was significantly higher than uninduced cultures and almost equal to periodontally affected MSCs. It has been known that proinflammatory mediators such as IL-1*β* and TNF-*α* play a crucial role in activating intracellular pathways required in cellular viability, proliferation, and differentiation toward certain cell lines. For instance, TNF-*α* induces the osteogenic differentiation of DPSCs by activation of the NF-*κ*B pathway [31, 40].

Besides, the inflammatory microenvironment activates cytoskeletal remodeling via interaction between high levels of chaperone proteins like heat shock protein 90 (hsp90), thioredoxin-1 (TXN-1), and heat shock protein A9 (hspA9) and actin filaments like cofilin, profiling, and vinculin, leading to stabilize actin filaments, and this interaction is closely related to the differentiation process [8]. Although the existence of an inflammatory state led to a decrease in the osteogenic differentiation in some studies [11, 32], GMSCs exhibited more resistance to inflammation-related changes compared to PDLSCs [11].

PDLSCs, on the other hand, have limited cell availability and reduced therapeutic viability, whereas gingival tissue is relatively abundant and easily accessible. Further, both MSCs from inflamed and noninflamed gingival tissues generated dense collagenous connective tissue resembles the natural gingiva [6, 7, 32], with type 1 collagen [6, 32], and expression of intrinsic cytokines MMP-1, MMP-2, IL-1, IL-6, and TNF-*α* were more evident in I-GMSCs [34]. Cross talk between GMSCs, extracellular matrix, inflammatory mediators, and inflammatory cells induce the profibrotic phenotype of cells.

A class of sensor proteins known as TLRs mediates recognition of PAMPs and initiation of inflammation as well as immune responses. Microbial products stimulate TLRs and lead to the activation of signaling pathways that result in the induction of antimicrobial genes and inflammatory cytokines. In addition to their expression in immune cells (e.g., dendritic cells and macrophages) and nonimmune cells (e.g., fibroblast and epithelial cells), several TLRs are expressed in MSCs and contribute to the protection against infection. Based on MSC origin, an inflammatory condition may modulate the pattern and role of TLRs expressed by MSCs [41, 42]. Among these MSCs, I-GMSCs highly express TLRs 1, 2, 4, 5, 7, and 10, enhancing the capacity of G-MSCs to recognize *in vivo* periodontal PAMPs [15].

Notably, H-GMSCs have the ability to ameliorate inflammation in a colitis experimental model, and I-GMSCs improve subcutaneous transplantation of murine skin allografts via activation or generation of infiltrating regulatory T cells [3, 6]. These may be attributed to highly conserved cross-species effects of cytokines, mediating the immunoregulatory effects of MSC or maybe due to the production of IL-10 and indoleamine 2,3-dioxygenase (IDO), which mediate immunoregulation [3, 6].

The migration of MSCs toward the damaged tissue is an important step in tissue regeneration. Concerning the migration capacity in the healthy and inflamed groups, there was no significant difference in the number of migrated GMSCs across different membrane micropore sizes, except for large sizes, where H-GMSCs displayed a higher migratory dynamic. This finding is related to developing selective driven membranes for tissue regeneration, particularly for periodontal regeneration, which enable stem cells to migrate through the scaffold [12]. IL-3 significantly enhances the migration, motility, and wound healing abilities of MSCs, including I-GMSCs, by upregulating the expression of chemokine receptors (CXCR4). Moreover, IL-3-induced CXCR4 expression leads to increased migration of MSCs towards SDF-1*α*, enhancing the migration and homing efficiency of MSCs [31].

Culture condition is one of the most essential factors in controlling MSC homing and migration efficiency: proinflammatory cytokines TGF-*β*1, IL-1*β*, and TNF-*α* facilitated BMSC migration across reconstituted human basement membranes by increasing production of MMP-2, MT1-MMP, and/or MMP-9 in these cells. This *in vitro* result provides a potential mechanism in MSC recruitment and migration toward the damaged tissue *in vivo* [43].

Lastly, I-GMSCs can be used as a useful and valued cell line for testing resin composite dental material due to the proximity of dental material to gingival tissue, and the standard cytotoxicity tests cannot predict *in vivo* behaviour of commercial materials. So, it is a good idea to determine the biocompatibility of dental materials at the cellular level using discarded human cell lines rather than animal lines [32].

## 5. Limitations

Included studies did not perform enough *in vitro* MSC characterization of cells derived from the inflamed tissue. Also, the lack of consistency between in vitro and subsequent *in vivo* study (ectopic formation) in the same article constituted another limitation in some studies.

## 6. Conclusion

The gingiva represents the easiest and most accessible MSC source in the oral cavity. Inflamed gingiva-derived MSCs are considered a unique biomedical waste as they retain their viability, morphology, phenotypic, and functional features of MSCs under inflammatory conditions. In addition to this, the inflammatory microenvironment promoted migration capacity and motility of GMSCs. Since the existence of an inflammatory microenvironment does not negatively affect MSC characterization, I-GMSCs could be used in experiments that involved diseases with persistent inflammatory conditions. In preclinical studies, I-GMSCs showed a colony formation ability similar or even more than healthy ones and a proliferation capacity faster than control groups. Besides, I-GMSCs could be a promising source in the following aspects: osteogenic differentiation, collagen fiber formation, immunoregulation, and testing of dental materials. Even though I-GMSCs represent a new therapeutic solution and could be a viable alternative source to H-GMSCs for tissue regeneration purposes, further and well-designed preclinical studies are required to confirm the previous findings and to determine the feasibility of this source. Equally important, the authors should follow the standard guideline as much as possible to reduce bias and gaps between preclinical searches and clinical use.

## Figures and Tables

**Table 1:** Characteristics and methodologies of included studies.

No.	Study ID	Study design	GMSC source	Sample collection		Experimental design			Scaffold or carrier (if present)
				Healthy	Diseased	Source of interest	Other sources (if present)	Control	
1	Tang et al., 2011 [6]	*In vitro* & *in vivo*	Human	Not specified	Gingivectomy due to drug-induced gingival hyperplasia	I-GMSCs	-	H-GMSCs H-BMSCs (*in vivo*)	Subcutaneous transplantation of stem cells loaded on fibrin gel

2	Ge et al., 2012 [7]	*In vitro* & *in vivo*	Human	Crown lengthening procedure	Flap debridement in chronic periodontitis	I-GMSCs	-	H-GMSCS H-PDLSCs (*in vivo*)	Subcutaneous transplantation of stem cells mixed with HA/TCP powder

3	Li et al., 2013 [34]	*In vitro* & in vivo	Human	Not specified	Gingivectomy due to dental plaque-induced gingival hyperplasia (*in vitro*) Cells were treated with culture containing inflammatory cytokines TNF-*α* and IL-1*β* (*in vivo*)	I-GMSCs (*in vitro*) Inflammatory cytokine-treated HGMSCs (*in vivo*)	Inflammatory cytokine-treated HDPSCs (*in vivo*)	H-GMSCs H-DPSCs	Subcutaneous transplantation of stem cell sheets

4	Yang et al., 2013 [11]	*In vitro* & in vivo	Human	Extraction of third molars	Cells were treated with culture containing inflammatory cytokines TNF-*α* and IL-1*β*	Inflammatory cytokine-treated HGMSCs	Inflammatory cytokine-treated HPDLSCs	H-GMSCS H-PDLSCs	Subcutaneous transplantation of stem cell loaded on artificial bone repair materials (iliac Golden®)

5	Fawzy El-Sayed et al., 2016 [15]	*In vitro*	Human	Free gingival collar	Cells were treated with culture containing inflammatory cytokines TNF-*α*, IL-1*β*, IFN-*γ*, and IFN-*α*	Inflammatory cytokine-treated HGMSCs	-	H-GMSCS	Culture medium

6	Barhanpurkar-Naik et al., 2017 [31]	*In vitro* and *in vivo*	Human	Not specified	Cells treated with inflammatory cytokine IL-3	Inflammatory cytokine-treated H-GMSCs	Inflammatory cytokine-treated HBMSCs and H-ADSCs	H-GMSCs H-BMSCs H-ADSCs	Culture medium

7	Tomasello et al., 2017 [8]	*In vitro*	Human	Extraction of third molars for orthodontic reasons	Extraction of periodontally affected molar	I-GMSCs Inflammatory cytokine-treated IGMSCs^∗^	I-DPSCs Inf. cytokine-treated HDPSCs (in some experiment)	H-GMSCS H-DPSCs	Culture medium

8	Zhang et al., 2017 [35]	*In vitro*	Human	Free gingival collar	Cells were treated with IL-1*β*, TNF-*α*, and IFN-*γ*	Inflammatory cytokine-treated H-GMSCs	Anti-inflammatory cytokine-treated H-GMSCs	H-GMSCs	Culture medium

9	Jauregui et al., 2018 [33]	*In vitro*	Human	Extraction of impacted third molars	Flap and osseous surgery in chronic periodontitis	I-GMSCs	_	H-GMSCs	Cells were seeded on electrospun polycaprolactone scaffolds

10	Soanca et al., 2018 [32]	*In vitro*	Human	Teeth extracted for orthodontic reasons	Gingival associated with overhanging posterior restoration	I-GMSCs	_	H-GMSCs	Culture medium

11	Yu et al., 2019 [16]	*In vitro* & *in vivo*	Mice	Gingival tissue from maxillary region	Ligature-induced periodontitis in mice	I-GMSCs	_	H-GMSCs	Systematic transplantation of stem cells (tail vein) into dextran sulfate sodium-induced mouse colitis

12	Al-Bahrawy et al., 2020 [12]	*In vitro*	Human	NS Author reported that discarded gingival sample from healthy gingiva	NS Author reported that discarded gingival sample from inflamed gingiva	I-GMSCs	_	H-GMSCs	Cells were seeded on perforated collagen-coated poly-tetra-floro-ethylene (PTFE) membrane

13	Cristaldi et al., 2020 [13]	*In vitro*	Human	Teeth extracted for orthodontic reasons	Extraction of periodontally affected teeth	I-GMSCs	_	H-GMSCs	Cells were seeded on Fisiograft Bone Granular® and Matriderm® scaffolds

**Table 2:** Results of included studies.

No.	Study ID	Clonogenic potential	Proliferation rate	Population doubling capacity	Phenotypic profile	Trilineage differentiation	Outcome measures	Method of investigation	Endpoint
1	Tang et al., 2011 [6]	I-GMSCs>H-GMSCs	Both sources proliferated similar to each other and faster than BMSCs	I-GMSCs>H-GMSCs>BMSCs	Similar expression/positive: STRO-1, CD29, CD44, CD90, CD105,CD146; negative: CD34, CD45	No significant difference (3 out 3) (osteo-adipo-chond)	Immunoregulation/immunosuppressive Formation of collagenous tissue	Coculture with T cell allogenic skin model Histological (H and E) and immunohistochemical analysis	Both cells showed increase ratio of regulatory T cells (Tregs) in comparison to BMSCs Both cells generated collagenous connective tissue with type 1 collagen was more evident in I-GMSCs
2	Ge et al., 2012 [7]	No significant difference	I-GMSCs>H-GMSCs	H-GMSCs>I-GMSCs	Similar expression/positive: CD44, CD73, CD90, CD105, CD166; negative: CD14, CD34, CD45	Similar capacity (3 out 3)	Formation of collagenous and mineralized tissues	Immunohistochemical analysis	Both GMSCs generated dense collagen tissue, while mineralized tissues observed only in PDLSCs
3	Li et al., 2013 [34]	No significant difference	I-GMSCs>H-GMSCs	Not reported^∗^	Similar expression/positive: CD29, CD90, CD44, CD105, STRO-1, CD146; negative: CD34, CD45	Different capacity (2 out 3) osteo+adipo (lower in I-GMSCs)	Osteogenic potential	Alzarin Red staining, ALP staining, RT-PCR	I-GMSCs exhibited lower adipogenic and osteogenic differentiation than H-GMSCs and osteogenic markers were heavily declined after treatment with cytokines
							Formation of collagenous tissue	Histological (H and E) RT-PCR	Inflammatory cytokine-treated GMSCs and DPSCs induced profibrotic phenotype. I-GMSCs had a higher expression of intrinsic cytokines (MMP)-1, MMP-2, IL-1, IL-6, TNF-*α*, and type 1 collagen than H-GMSCs
4	Yang et al., 2013 [11]	Compare between healthy groups (H-GMSCs and H-PDLSCs)	Compare between healthy groups (H-GMSCs and H-PDLSCs)	Compare between healthy groups (H-GMSCs and H-PDLSCs)	Compare between healthy groups (H-GMSCs and H-PDLSCs)	Compare between healthy groups (H-GMSCs and H-PDLSCs)	Osteogenic potential	Alizarin Red staining, ALP staining, and RT-qPCR/expression of osteogenic markers (OCN, RUNX2, and COL1)	Both cells showed decline in the osteogenic potential, more significantly in PDLSCs compared to GMSCs
							Formation of bone	Histological (H and E, MT staining)	Inflammatory cytokine-treated cells showed decrease in bone formation capacity in comparison to the untreated ones, higher osteogenic area in inf. cytokine-treated PDLMSCs than GMSCs.
5	Fawzy El-Sayed et al., 2016 [15]	# done for H-GMSCs	# done for HGMSCs	ND	# done for H-GMSCs	#done for HGMSCs (3 out 3) (osteo-adipo-chond)	TRL expression profile	Flow cytometry, mRNA expression	H-GMSCs expressed TLRs 1, 2, 3, 4, 5, 6, 7, and 10, while inf. cytokine-treated GMSCs expressed TLRs 1, 2, 4, 5, and 7 as well as 10 without TLR 6
6	Barhanpurkar –Naik et al., 2017 [31]	ND	ND	ND	NR # done for H-MSCs, but data not presented	ND	IL-3R*α* expression profile	Flow cytometry, mRNA expression	The mRNA expression of 3 sources was similar; however, the protein expression was different (BMSCs and ADSCs showed higher expression of IL-3R*α* than GTMSCs)
							Migration, motility, and wound healing capacity	In vitro cell migration, motility, and wound healing assays	No significant differences in all three sources; IL-3 enhanced migration, motility, and wound closure.
7	Tomasello et al., 2017 [8]	Significant difference I-GMSCs and I-DPSCs>H-GMSCs and H-DPSCs	I-DPSCs and I-GMSCs>H-DPSCs and H-GMSCs	I- GMSCs and I-DPSCs>H-GMSCs and H-DPSCs	Slight different expression/positive: STRO-1, CD146, CD29, SSEA4 (higher expressed in diseased groups); negative: CD34, CD45, HLA-DR	ND	Osteogenic potential	Alizarin Red staining and RT-qPCR/expression of osteogenic markers (RUNX2, OPN, and OCN)	Inflammatory cytokine-treated H-DPSCs and H-GMSCs as well as I-MSCs were deeply stained and highly expressed osteogenic markers than untreated H-MSCs.
8	Zhang et al., 2017 [35]	Significant difference H-GMSCs>I-GMSCs and anti-IGMSCs	Various proliferation rate according to duration^∗^; (last time point 12 day) H-GMSCs>I-GMSCs and anti-IGMSCs	ND	# done for H-GMSCs	#done for HGMSCs (3 out 3) (osteo-adipo-chond)	Osteogenic potential	Alizarin Red staining and RT-qPCR/expression of osteogenic markers (RUNX2, ALP, COL1, and OPN)	The number of calcified nodules as well as expression of osteogenic factors were higher in the anti-inflammatory group than in the control group and in the inflammatory group
9	Jauregui et al., 2018 [33]	H-GMSCs>I-GMSCs	Both sources exhibited increase in proliferation rate	ND	Similar expression/positive: CD105, CD73, CD90; negative: CD19, CD34, CD45, CD11b, HLA-DR	Different capacity (2 out of 3) H-GMSCs had higher osteogenicity, while I-GMSCs showed increased adipogenesis	Osteogenic potential	Alizarin Red staining and ALP activity	Both cell sources had a tendency toward osteogenic differentiation.
10	Soanca et al., 2018 [32]	ND	Both sources exhibited Increase in proliferation rate	ND	Similar expression/positive: CD105, CD73, CD90, CD49e, CD29, CD44, CD166; negative: CD14, CD34, CD45, CD79, CD117, HLA-DR	Similar capacity with few cells expressed osteogenic markers (3 out 3+neurogenic).	Testing biocompatibility of resin composite	PKH26 cell membrane fluorescent labeling MTT assay	I-GMSCs can be used as valuable cell line for testing dental material
11	Yu et al., 2019[16]	H-GMSCs>I-GMSCs	H-GMSCs>I-GMSCs	H-GMSCs>I-GMSCs	Different expression/positive: Sca1, CD105, CD90, CD73 (significantly decreased in IGMSCs); negative: CD45, CD34	I-GMSCs showed decrease tridifferentiation potential	Immunomodulation	Coculture with T cells	I-GMSCs showed impaired immunomodulatory properties, and this impairment was rescued when cells treated with acetylsalicylic acid.
12	Al- Bahrawy et al., 2020 [12]	No significant differences	Both sources exhibited increase in proliferation rate	I-GMSCS>H-GMSCs	Positive: CD105, CD73, CD90, CD164; negative: CD14, CD34, CD45	Similar capacity (3 out 3)	Migration capacity	Migration assay	No significant difference between I-GMSCs and H-GMSCs through small pores, while H-GMSCs migrated better than I-GMSCs through large-pore membrane
13	Cristaldi et al., 2020 [13]	I-GMSCs>H-GMSCs	I-GMSCs>H-GMSCs	I-GMSCS>H-GMSCs	Slight different expression/positive: CD73, CD29, CD90, CD105 (slightly increased expression of CD90 and CD105 in IGMSCs); negative: CD45, HLA-DR	(1 out 3) osteogenic	Cell growth	Different cell viability assay	Cell growth or colonization on collagen scaffold was higher in I-GMSCs than H-GMSCs
							Osteogenic potential	Alizarin Red staining and RT-qPCR/expression of osteogenic markers (RUNX2, OCN, and OPN)	Both GMSCs did not grow on nano HA, while they grew effectively on collagen scaffold compared to the unloaded cells/both cells showed a moderate expression of osteogenic markers (RUNX2, OCN, OPN)

## Abbreviations

ADSCs: Adipose-derived stem cells
ALP: Alkaline phosphatase
ASA: Acetylsalicylic acid
BMSCs: Bone marrow mesenchymal stem cells
CFU: Colony-forming units
COL1: Collagen type 1
CXCR: Chemokine receptor
DPSCs: Dental pulp stem cells
FASL: Fas Ligand
GMSCs: Gingival mesenchymal stem cells
HA: Hydroxyapatite
H-GMSCs: Healthy gingiva-derived mesenchymal stem cells
hsp: Heat shock protein
IDO: Indoleamine 2,3-dioxygenase
IFN-*γ*: Interferon-*γ*
I-GMSCs: Inflamed gingiva-derived mesenchymal stem cells
IL-1*β*: Interleukin-1*β*
ISCT: International Society for Cellular Therapy
MMP: Matrix metallopeptidase
MSCs: Mesenchymal stem cells
NSAID: Nonsteroidal anti-inflammatory drug
OCN: Osteocalcin
OPN: Osteopontin
PAMPs: Pathogen-associated molecular patterns
PDLSCs: Periodontal ligament mesenchymal stem cells
RUNX2: Runt-related transcription factor 2
TLRs: Toll-like receptors
TNF-*α*: Tumor necrosis factor-*α*
Tregs: Regulatory T cells
TXN-1: Thioredoxin-1.

## Supplementary Materials

Supplementary Materials Supplementary material I: the quality of the *in vitro* and *in vivo* studies selected in this scoping review.
