# Supplementary material for: The Therapeutic Potential of Inflamed Gingiva-Derived Mesenchymal Stem Cells in Preclinical Studies: A Scoping Review of a Unique Biomedical Waste
Source: Stem Cells Int. 2021 Feb 10;2021:6619170. doi: 10.1155/2021/6619170 (PMC7889391; doi:10.1155/2021/6619170)
Supplement: Supplementary Materials — Supplementary material I: the quality of the in vitro and in vivo studies selected in this scoping review. [file 6619170.f1.pdf]

Table 1: The quality of selected in vitro studies (modified from the ARRIVE and CONSORT guidelines) (Faggion, 2012; Ramamoorthi et al., 2015).

Item	Description	Grade	Fawzy El-Sayed et al., 2016	Tomasello et al., 2017	Zhang et al., 2017	Jauregui et al., 2018	Soanca et al., 2018	Al-Bahrawy et al., 2020	Cristaldi et al., 2020
1	Title	(0) Inaccurate/non concise<br>(1) Concise/adequate	1	1	1	1	1	1	1
2	Abstract: either a structured summary of background, research objectives, key experiment methods, principal findings, and conclusion of the study or self-contained (should contain enough information to enable a good understanding of the rationale for the approach).	(1) Clearly inadequate<br>(2) Possibly accurate<br>(3) Clearly accurate	3	3	3	3	3	3	3
3	Introduction: background, experimental approach, and explanation of rationale/hypothesis.	(1) Insufficient<br>(2) Possibly sufficient/some information<br>(3) Clearly sufficient	3	3	3	3	3	3	3
4	Introduction: preprimary and secondary objectives for the experiments (specific primary/secondary objectives).	(1) Not clearly stated<br>(2) Clearly stated	2	2	2	2	2	2	2
5	Methods: study design explained number of experimental and control groups, steps to reduce bias (demonstrating the consistency of the experiment (done more than once), sufficient detail for replication, blinding in evaluation, etc.).	(1) Clearly insufficient<br>(2) Possibly sufficient<br>(3) Clearly sufficient	1	2	3	2	1	2	2
6	Methods: precise details of experimental procedure (i.e., how, when, where, and why).	(1) Clearly insufficient<br>(2) Possibly sufficient<br>(3) Clearly sufficient	3	3	2	3	2	3	3
7	Methods: How sample size was determined (details of control and experimental group) and sample size calculation.	(1) No<br>(2) Unclear/not complete<br>(3) Adequate/clear	2	2	2	2	2	2	2
8	Methods: Details of statistical methods and analysis (statistical methods used to compare groups).	(1) No<br>(2) Unclear/not complete<br>(3) Adequate/clear	3	3	3	3	3	3	3
9	Results: explanation for any excluded data, results of each analysis with a measure of precision as standard	(1) No<br>(2) Unclear/not complete<br>(3) Adequate/clear	2	3	2	2	2	2	2

	deviation or standard error or confidence interval.								
10	Discussion: interpretation/scientific implication, limitations, and generalizability/translation.	(1) Clearly inadequate<br>(2) Possibly accurate<br>(3) Clearly accurate	3	3	3	3	2	2	3
11	Statement of potential conflicts and funding disclosure	(0) No<br>(1) Yes	0	1	1	1	1	0	1
12	Publication in a peer-review journal	(0) No<br>(1) Yes	1	1	1	1	1	1	1

Table 2: The quality of selected in vivo studies (modified from the ARRIVE guidelines) (Kilkenny et al., 2010; Ramamoorthi et al., 2015).

Item	Description	Grade	Tang et al., 2011	Ge et al., 2012	Li et al., 2013	Yang et al., 2013	Barhanpurkar-Naik et al., 2017	Yu et al., 2019
1.	Title	(0) Inaccurate/non concise<br>(1) Concise/adequate	1	1	1	1	1	1
2.	Abstract: either a structured summary of background, research objectives, key experiment methods, principal findings, and conclusion of the study or self-contained (should contain enough information to enable a good understanding of the rationale for the approach).	(1) Clearly inadequate<br>(2) Possibly accurate<br>(3) Clearly accurate	3	3	2	3	3	3
3.	Introduction: background, experimental approach, and explanation of rationale/hypothesis.	(1) Insufficient<br>(2) Possibly sufficient/some information<br>(3) Clearly sufficient	2	3	3	3	3	3
4.	Introduction: preprimary and secondary objectives for the experiments (specific primary/secondary objectives).	(1) Not clearly stated<br>(2) Clearly stated	1	2	1	2	2	1
5.	Method: ethical statement for the use of animal	(1) Clearly insufficient<br>(2) Possibly sufficient<br>(3) Clearly sufficient	3	3	3	3	3	3
6.	Methods: study design explained number of experimental<br>and control groups,<br>• In vitro: steps to reduce bias (demonstrating the consistency of the experiment (done more than once), sufficient detail for replication, blinding in evaluation, etc.).<br>• In vivo: steps to reduce bias by allocation concealment, randomization, and binding	(1) Clearly insufficient<br>(2) Possibly sufficient<br>(3) Clearly sufficient	1	2	2	2	2	2
7.	Methods: precise details of experimental procedure (i.e., how, when, where, and why).	(1) Clearly insufficient<br>(2) Possibly sufficient<br>(3) Clearly sufficient	3	3	3	2	3	3
8.	Methods: experimental animal species, strains, sex, development stage, weight, and source of animals, housing and husbandry conditions (welfare related	(1) Clearly insufficient<br>(2) Possibly sufficient<br>(3) Clearly sufficient	1	1	1	1	1	1

	assessments and interventions include type of cage, bedding material, number of cage companions, temperature, light or dark cycle, and access to food and water)							
9.	Methods: How sample size was determined (details of control and experimental group) and sample size calculation.	(1) No<br>(2) Unclear/not complete<br>(3) Adequate/clear	1	1	1	1	1	1
10.	Methods: outcomes (clearly defines the experimental methods to evaluate the pre-specified outcomes)	(1) No<br>(2) Unclear/not complete<br>(3) Adequate/clear	3	3	3	3	3	3
11.	Methods: Details of statistical methods and analysis (statistical methods used to compare groups).	(1) No<br>(2) Unclear/not complete<br>(3) Adequate/clear	3	3	3	3	3	3
12.	Results: baseline data (characteristic and health status of animals), explanation for any excluded data, results of each analysis with a measure of precision as standard deviation or standard error or confidence interval.	(1) No<br>(2) Unclear/not complete<br>(3) Adequate/clear	1	2	2	1	2	1
13.	Discussion: interpretation/scientific implication, limitations, and generalizability/translation.	(1) Clearly inadequate<br>(2) Possibly accurate<br>(3) Clearly accurate	3	3	3	3	3	3
14.	Statement of potential conflicts and funding disclosure	(0) No<br>(1) Yes	0	1	1	0	1	1
15.	Publication in a peer-review journal	(0) No<br>(1) Yes	1	1	1	1	1	1
